# Gastrointestinal microbiota alterations associated with pediatric gastroesophageal reflux disease and proton pump inhibitor exposure: a systematic review of microbiome changes, dysbiosis markers, and associated clinical outcomes

**DOI:** 10.3389/fnut.2026.1909690

**Published:** 2026-08-12

**Authors:** Vasile Valeriu Lupu, Elena Jechel, Anca Adam Raileanu, Sorana Caterina Anton, Laura Iulia Bozomitu, Otilia Elena Frasinariu, Oana Raluca Temneanu, Lorenza Forna, Magdalena Cuciureanu, Alin Horatiu Nedelcu, Carmen Rodica Anton, Elena Cristina Mitrofan, Diana Elena David, Daniela Carmen Rusu, Dragos Munteanu, Alexandru Vlase, Emil Anton, Ancuta Lupu

**Affiliations:** 1 Grigore T. Popa University of Medicine and Pharmacy, Iasi, Romania; 2 Clinic of Pulmonary Disease, Iasi, Romania

**Keywords:** aerodigestive tract microbiome, children, dysbiosis, gastroesophageal reflux disease, gut microbiota, microbiome alterations, pediatric microbiome, proton pump inhibitors

## Abstract

**Introduction:**

Gastroesophageal reflux disease (GERD) and proton pump inhibitor (PPI) therapy have both been associated with alterations of the developing gastrointestinal microbiome and potential downstream clinical consequences in children. However, available evidence is fragmented across disease-related, treatment-related, and mechanistic studies, making the overall biological relationship difficult to interpret.

**Methods:**

PubMed, Web of Science, and Scopus databases were queried for the period January 2000 to May 2026 to synthesize evidence across the pathophysiological continuum linking pediatric GERD, microbiota alterations, inflammatory mechanisms, PPI exposure, and microbiota-associated clinical outcomes.

**Results:**

The review included studies evaluating children with GERD as well as mechanistic studies involving pediatric upper gastrointestinal conditions requiring PPI therapy when microbiota-related outcomes were relevant to the review objective. Study selection was performed according to PRISMA guidelines, 31 of which were included in the final analysis. The data indicate that pediatric exposure to GERD and PPI is associated with changes in the gastrointestinal microbiota at multiple levels (oral, esophageal, gastric, and intestinal). These changes include reduced microbial diversity, changes in bacterial composition (particularly *Firmicutes*, *Proteobacteria*, *Bacteroidetes*, and *Actinobacteria*), and increased similarity of microbiota between aerodigestive compartments, suggesting possible microbial transfer associated with reflux or aspiration. PPI exposure has been frequently associated with increased numbers of oropharyngeal bacteria in the gut, decreased beneficial taxa (e.g., *Bifidobacterium*), and increased numbers of potentially pathogenic genera (e.g., *Streptococcus*, *Enterobacteriaceae*). Several studies have reported an association between PPI and small intestinal bacterial overgrowth, but the results are inconsistent. PPI use has also been reported to be associated with an increased risk of infections, allergic diseases, and other systemic effects, coupled with the recognition of limited clinical benefit of PPI therapy in GERD in infants.

**Conclusion:**

Pediatric GERD and PPI exposure are associated with alterations in the gastrointestinal microbiome and possible systemic clinical effects. Most evidence is heterogeneous and influenced by confounding factors, which limits the establishment of a causal relationship. Prospective longitudinal and mechanistic studies using standardized microbiome methodologies are required to clarify causality and better define the respective contributions of GERD and PPI therapy to microbiota alterations and their clinical significance.

**Systematic review registration:**

https://www.crd.york.ac.uk/PROSPERO/view/CRD420261405291, identifier CRD420261405291.

## 1 Introduction

Gastroesophageal reflux (GER) is the retrograde passage of gastric contents into the esophagus and is a common physiological phenomenon in infancy. Regurgitation occurs in approximately 50% of healthy infants in the first months of life, reaching a peak around 4 months of age and spontaneously remitting in most children by 12–18 months of age. This phenomenon is favored by the functional immaturity of the lower esophageal sphincter, the predominantly liquid diet and the horizontal position characteristic of the infant. In contrast, gastroesophageal reflux disease (GERD) is defined by the occurrence of bothersome symptoms and/or complications caused by GER, affecting the child’s health and quality of life. Reflux esophagitis refers to endoscopically and/or histologically documented esophageal mucosal injury secondary to gastroesophageal reflux and represents one of the recognized complications of GERD (1, 2). The prevalence of GERD in the pediatric population varies considerably depending on age, definitions used, and assessment methods. A systematic review by Singendonk et al. reported a prevalence of reflux symptoms ranging from 0 to 38% in children and adolescents, with GER being one of the most common reasons for presentation to pediatricians and pediatric gastroenterologists (3). For this reason, GERD is an important reason for prescribing antisecretory therapy in pediatric practice (4).

Proton pump inhibitors (PPI) are currently the main therapeutic class used for acid suppression in pediatric GERD. However, their use has increased significantly in the last two decades, including in infants in whom the diagnosis of GERD is often difficult to differentiate from physiologic reflux. Several studies and systematic reviews have demonstrated that symptoms such as excessive crying, irritability, sleep disturbance, or frequent regurgitation do not reliably predict the presence of acid reflux and do not consistently respond to PPI therapy, suggesting the existence of an overprescription phenomenon in this age group (2, 5).

Traditionally, the pathophysiology of GERD has been explained by the “acid-centric” model, according to which gastric acid is the main factor responsible for the onset of symptoms and esophageal damage. However, experimental and clinical data accumulated in recent years suggest that esophageal inflammation involves more complex biological mechanisms, including alteration of the epithelial barrier, activation of the immune response, and release of proinflammatory cytokines, processes that cannot be explained exclusively by direct chemical aggression of gastric acid (6, 7). These observations have contributed to the reevaluation of the classical paradigm of GERD and the development of more complex pathophysiological models. In parallel, the progress of genomic sequencing technologies has allowed the characterization of the digestive tract microbiota and highlighted its role in maintaining gastrointestinal homeostasis. Recent studies have demonstrated that the esophagus is not a sterile organ, but hosts specific microbial communities that differ between healthy individuals and those with reflux disease or esophagitis. These observations have led to the emergence of the concept of the “gut–esophagus axis,” which assumes the existence of bidirectional interactions between the intestinal microbiota, the esophageal microbiota and the host immune response (8, 9). In this context, changes in microbiota are considered potential factors involved in both the pathogenesis of GERD and the response to treatment.

The development of the gut microbiota begins in the perinatal period and continues in a dynamic process throughout the first years of life. The mode of delivery is one of the main factors shaping the composition of the neonatal microbiota. Newborns born vaginally are colonized predominantly with maternal microorganisms from the vaginal and intestinal microbiota, while children born by cesarean section show delayed colonization with species such as *Bacteroides* and *Bifidobacterium* and a different microbial diversity in the first months of life (10, 11). Nutrition is another major determinant of microbiome development. Breast milk favors the development of a microbiota dominated by bifidobacteria due to its rich content of specific oligosaccharides and bioactive factors with immunomodulatory roles. In contrast, formula feeding results in a distinct microbial composition and bacterial diversity different from that observed in exclusively breastfed infants (12, 13). Subsequently, the introduction of complementary feeding induces important changes in the structure of the microbiota, favoring the progressive transition toward a microbial profile similar to that found in adults (14).

Antibiotic exposure during the neonatal period and early childhood is one of the most important factors disrupting the gut microbiota. Early administration of antibiotics can cause a reduction in bacterial diversity, altered colonization with beneficial microorganisms, and persistent changes in the gut microbial ecosystem, effects later associated with immunological and metabolic disorders (15, 16). Similarly, the use of PPI can alter the composition of the microbiota by increasing gastric pH and reducing the function of the physiological acid barrier, favoring the survival and colonization of microorganisms that would normally be eliminated at the gastric level (17, 18).

Currently, the first 2 to 3 years of life are considered a critical period for microbiota maturation and immune system development. During this stage, the establishment of the main intestinal microbial communities and the development of immunological tolerance take place, and disruptions caused by factors such as cesarean section, artificial feeding, antibiotics or PPI can have long-term consequences on digestive and extraintestinal health (19, 20).

## 2 Materials and methods

Recent evidence suggests that pediatric GERD may involve gut microbial alterations and immune-mediated mechanisms, while proton pump inhibitor exposure may further exacerbate dysbiosis and influence long-term health outcomes. However, current literature is heterogeneous, with variations in study design, patient populations, microbiome assessment methods, and reported outcomes, making it difficult to draw consistent conclusions regarding the nature and clinical relevance of these findings. Importantly, the available evidence is distributed across complementary domains, including disease-associated microbiota alterations, microbiota-related inflammatory mechanisms, PPI-associated microbial changes, and potential downstream clinical consequences. Although these domains have often been investigated separately, they represent biologically interconnected components of the same pathophysiological continuum. Therefore, the primary objective of this systematic review was to synthesize the available evidence across this continuum, integrating disease-related and treatment-related microbiota alterations in order to better understand their potential contribution to pediatric GERD pathophysiology and associated clinical outcomes. This study was conducted according to the PRISMA guidelines (21). The study protocol was registered PROSPERO, registration No. CRD420261405291.

The review was designed around a single overarching research question: how alterations of the gastrointestinal microbiota contribute to the pathophysiology of pediatric GERD and how proton pump inhibitor therapy may further influence microbial composition and microbiota-associated clinical outcomes. To comprehensively address this objective, evidence was synthesized across predefined but biologically interconnected domains, including disease-associated microbiota alterations, inflammatory and epithelial barrier mechanisms, PPI-associated microbiota changes, and their potential clinical implications. These domains were considered complementary components of the same conceptual framework rather than independent clinical questions.

### 2.1 Search strategies

A systematic literature search was performed using the electronic databases PubMed/MEDLINE, Web of Science, and Scopus. The search covered the period from January 2000 to May 2026 in order to capture the emergence and evolution of microbiome-related research in pediatric gastroenterology. The search strategy combined controlled vocabulary terms (MeSH where applicable) and free-text keywords related to pediatric gastroesophageal reflux disease (GERD), gastrointestinal microbiota alterations, inflammatory and immune-related mechanisms, and proton pump inhibitor (PPI) exposure.

To ensure a comprehensive and structured identification of relevant studies, four complementary search levels were applied according to the syntax requirements of each database. The first search level focused on pediatric GERD and microbiota-related alterations: ((“Gastroesophageal Reflux”[Mesh] OR “gastroesophageal reflux disease” OR GERD OR reflux esophagitis OR NERD)) AND (“Microbiota”[Mesh] OR microbiome OR “gut microbiota” OR dysbiosis OR “intestinal microbiota”) AND (child* OR pediatric* OR infant* OR adolescent*). The second search level explored the association between GERD, microbiota changes, and PPI exposure: (GERD OR “gastroesophageal reflux disease”) AND (microbiome OR microbiota OR dysbiosis) AND (“proton pump inhibitor” OR PPI OR omeprazole OR esomeprazole OR lansoprazole) AND (pediatric* OR child* OR infant*). The third search level investigated microbiota-associated inflammatory and mucosal mechanisms in pediatric GERD: (GERD OR “gastroesophageal reflux disease”) AND (microbiome OR microbiota OR dysbiosis) AND (“inflammation” OR cytokines OR “immune activation” OR “epithelial barrier dysfunction” OR “mucosal inflammation”) AND (child* OR pediatric*). The fourth search level focused on pediatric PPI exposure and microbiota alterations independently of GERD-specific diagnosis, in order to capture mechanistic and treatment-related evidence relevant to upper gastrointestinal disorders: (“proton pump inhibitor” OR PPI OR omeprazole OR esomeprazole OR lansoprazole) AND (microbiome OR microbiota OR dysbiosis OR “gut microbiota”) AND (infant* OR child* OR pediatric* OR adolescent*). Reference lists of included studies and relevant reviews were additionally screened manually to identify potentially eligible articles not retrieved through the initial database search.

Although four complementary search domains were applied, these were predefined to identify evidence addressing different aspects of the same biological framework rather than separate research questions. Because microbiota alterations observed in pediatric GERD may reflect both disease-associated mechanisms and treatment-related effects, studies evaluating PPI-associated microbiota changes in other pediatric upper gastrointestinal disorders were also considered when they provided mechanistic or microbiota-related evidence relevant to pediatric GERD. Likewise, studies investigating inflammatory pathways and epithelial barrier dysfunction were included to facilitate the biological interpretation of microbiota alterations observed in pediatric GERD.

Studies investigating microbiota alterations in pediatric gastroesophageal reflux disease (GERD), microbiota-associated inflammatory pathways, epithelial barrier dysfunction, or long-term clinical outcomes potentially associated with microbiota disruption or proton pump inhibitor exposure were considered eligible for inclusion. Studies evaluating PPI-associated microbiota changes in reflux-associated esophageal disorders, were also considered when relevant to the review objectives and microbiome-related outcomes. We excluded meta-analyses, narrative reviews, editorials, letters to the editor, conference abstracts without full text, practice guidelines, encyclopedias, animal-only studies, and studies exclusively involving adult populations. Reference lists of relevant articles were manually screened to identify additional eligible studies potentially missed during the database search. Furthermore, proceedings and digital archives from major pediatric gastroenterology scientific meetings and international registries were consulted to identify unpublished or ongoing studies relevant to the topic. To minimize the risk of publication bias and omission of potentially relevant data, no language restrictions were applied during the initial screening process.

### 2.2 Study selection criteria

The inclusion of studies was carried out according to the PICOS framework (Table 1). The review was designed to address a single overarching objective: to synthesize the available evidence regarding microbiota alterations throughout the pathophysiological continuum of pediatric GERD, integrating disease-associated microbial changes, microbiota-related inflammatory mechanisms, microbiota modulation associated with proton pump inhibitor therapy, and microbiota-related clinical outcomes. Rather than representing separate research questions, these domains were considered complementary components of a single conceptual framework describing the interaction between pediatric GERD, microbiota, and acid suppression therapy. Accordingly, the review sought to answer the following overarching research question: “What is the current evidence supporting the role of microbiota alterations in pediatric GERD, and how may proton pump inhibitor therapy further modify the developing microbiome and contribute to microbiota-associated clinical outcomes?” Secondary objectives included evaluating whether microbiota alterations are consistently associated with pediatric GERD, whether PPI exposure is associated with microbial dysbiosis, and which inflammatory, immune-related, infectious, allergic, neurodevelopmental, metabolic, and systemic outcomes have been associated with microbiota disruption following acid suppression therapy.

**TABLE 1:** PICOS table.

PICOS Element	Description
Population (P)	Children (0–18 years) with GERD or reflux esophagitis. Studies involving other pediatric upper gastrointestinal disorders requiring PPI therapy were included only when they reported microbiota-related or mechanistic findings directly relevant to understanding microbiota alterations associated with pediatric GERD and acid suppression therapy.
Intervention / Exposure (I)	Disease-associated microbiota alterations in pediatric GERD and microbiota modulation associated with PPI therapy, evaluated in relation to inflammatory mechanisms, epithelial barrier dysfunction, and microbiota-associated clinical outcomes. These exposures were considered biologically interconnected components of the same pathophysiological framework.
Comparator (C)	Healthy pediatric controls, children without GERD, children not exposed to PPI, or within-study comparator groups defined according to the individual study design (e.g., treated vs. untreated, different microbiota profiles, or clinical phenotypes). Studies without an explicit comparator group were also considered when they provided mechanistic or microbiota-related evidence relevant to the review objective.
Outcomes (O)	Primary outcomes: changes in gastrointestinal microbiota composition, microbial diversity, differential abundance of bacterial taxa, dysbiosis, markers of mucosal inflammation, epithelial barrier dysfunction, and immune activation. Secondary outcomes: clinical disease severity, treatment response, SIBO, and long-term infectious, allergic, metabolic, neurodevelopmental, and other microbiota-associated systemic outcomes potentially related to GERD and/or PPI exposure.
Study design (S)	Observational and interventional human studies, including cohort, case-control, cross-sectional, longitudinal, and clinical trial designs, evaluating microbiota-related findings in pediatric GERD or PPI-treated pediatric upper gastrointestinal disorders. Narrative reviews, systematic reviews, editorials, expert opinions, conference abstracts without full text, animal studies, and studies involving exclusively adult populations were excluded.

GERD, gastroesophageal reflux disease; PPI, proton pump inhibitor; SIBO, small intestinal bacterial overgrowth.

Eligible studies included pediatric populations (0–18 years) diagnosed with GERD or reflux esophagitis. Studies involving other pediatric upper gastrointestinal disorders requiring proton pump inhibitor therapy were considered only when they reported microbiota-related outcomes or mechanistic findings directly relevant to understanding PPI-associated microbiota modulation within the overall conceptual framework of pediatric GERD. Observational studies, cohort studies, case-control studies, cross-sectional studies, clinical trials, and mechanistic human studies were considered eligible for inclusion.

After the identification and centralization of all retrieved records, the studies were independently screened by two investigators using a standardized data extraction form. The selection process was conducted in several stages: first by reviewing the titles, subsequently the abstracts, and finally the full-text articles considered potentially relevant. Studies not fulfilling the predefined eligibility criteria were excluded. The extracted data included the following variables: authors, year of publication, country of origin, study design, sample size, age group, diagnostic criteria for GERD, characteristics of PPI exposure, methods used for microbiota analysis, reported microbial taxa alterations, inflammatory markers, measures of microbial diversity, clinical outcomes, and the main conclusions of each study. Particular attention was given to reported alterations in bacterial populations, as well as to markers of low-grade mucosal inflammation and epithelial barrier dysfunction. Any disagreements between the two reviewers regarding study eligibility or data extraction were resolved through discussion and consensus with a third investigator.

A meta-analysis was not planned because substantial clinical and methodological heterogeneity was anticipated regarding study design, participant characteristics, microbiome assessment methods, exposure definitions, and reported outcomes. Therefore, findings were synthesized narratively. The selected studies were analyzed comparatively in order to identify recurring microbiota patterns, potential inflammatory and microbiota-related mechanisms, and the potential implications of prolonged PPI exposure during critical periods of microbiome development in childhood. To complement the methodological quality assessment, the certainty of the evidence was evaluated using the Grading of Recommendations Assessment, Development and Evaluation (GRADE) approach. Because no quantitative meta-analysis was performed and the included studies were highly heterogeneous with respect to design, populations, microbiome assessment methods, and reported outcomes, the GRADE assessment was conducted narratively for the main outcome domains rather than on pooled effect estimates. Outcome domains were predefined according to the objectives of the review and included GERD-associated microbiota alterations, PPI-associated microbiota alterations, small intestinal bacterial overgrowth (SIBO)/dysbiosis-related small bowel alterations, PPI efficacy and pharmacokinetics, allergic outcomes, infectious outcomes, neuropsychiatric outcomes, and epilepsy/cancer-related outcomes. For each outcome, the certainty of the evidence was assessed across the standard GRADE domains (risk of bias, inconsistency, indirectness, and imprecision), resulting in an overall certainty rating of high, moderate, low, or very low.

## 3 Results

**FIGURE 1:**
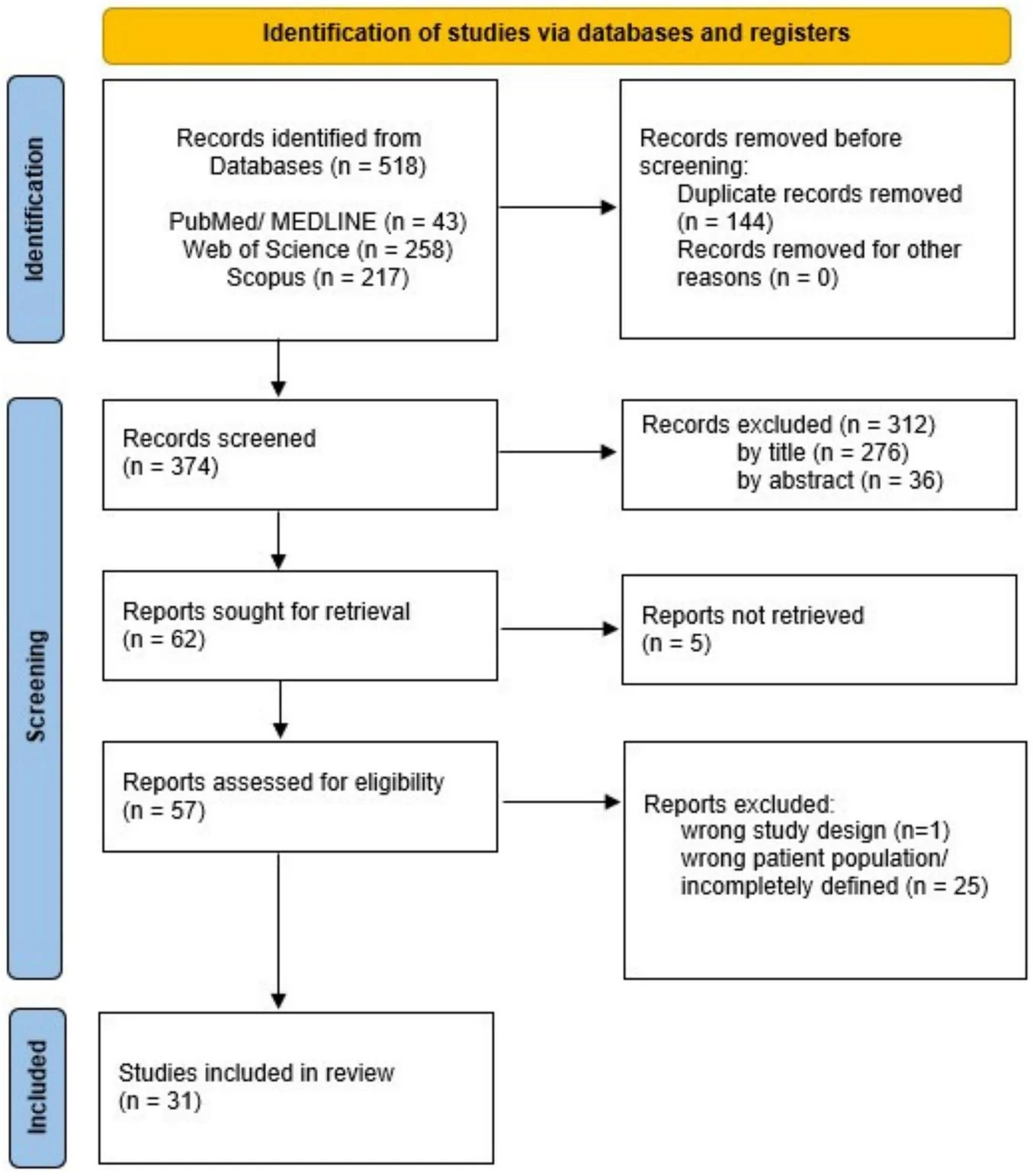
PRISMA scheme regarding the search strategy and its results.

A total of 518 studies were identified after the general search. The data were entered into the Zotero bibliographic manager. After excluding duplicates (*n* = 144), the total number was reduced to 374 items. Of these, 312 studies were excluded because they were considered unrelated to the subject, and in the case of 5 studies, the full form of the article could not be retrieved. Of the remaining total (*n* = 57), after applying the inclusion and exclusion criteria, 31 articles were considered eligible (22–52). The full text of each of the selected studies was read and analyzed to confirm eligibility. Because one of the objectives of this review was to explore the potential clinical consequences associated with microbiota alterations during PPI therapy, studies evaluating clinical outcomes of PPI exposure (e.g., safety, pharmacokinetics, and long-term adverse outcomes) were also included, even when they did not directly assess the microbiome. These studies were analyzed separately from those providing direct microbiome data and were interpreted as indirect evidence only. The graphic illustration of the process of identification, screening and validation of reference works according to the PRISMA criteria is shown in Figure 1. The main characteristics of the included studies are summarized in Table 2, while the methodological quality and risk of bias assessment for each included study are presented separately in Table 3.

**TABLE 2:** Summarizing systematized articles.

Article	Purpose/ Objective	Intervention/Exposure	Comparator	Outcomes evaluated
Duvallet et al. (22)	Evaluation of microbiome exchange between the oropharynx, stomach and lung in children with impaired swallowing function and the role of reflux/aspiration in changes in the pulmonary microbiome	Collection of bronchoalveolar, gastric and oropharyngeal samples; 16S rRNA sequencing; evaluation of reflux and swallowing function	Children with oropharyngeal aspiration vs no aspiration; Comparisons regarding the presence of gastroesophageal reflux	Association between reflux/aspiration and changes in the pulmonary microbiome
Boers et al. (23)	Investigation of the effect of gastroesophageal reflux disease (GER) on the nasopharyngeal microbiome and middle ear in children with otitis media	Taking samples from the fluid of the middle ear and nasopharynx; microbiome analysis by 16S rRNA sequencing	Children with GER vs children without GER	Composition of the nasopharyngeal and middle ear microbiome; the presence of pathogenic bacteria (e.g., *Haemophilus, Streptococcus, Alloiococcus*); association of GER with microbial changes
Harris et al. (24)	Investigation of the esophageal microbiome in EoE and evaluation of differences from GERD and healthy subjects	Esophageal String Test and microbiome analysis by 16S rRNA sequencing	EoE vs GERD vs. Healthy Controls	Esophageal bacterial load; the composition of the microbiome; Differences between groups
Luu et al. (25)	Evaluation of the differences between oral, esophageal and gastric microbiome in children and the impact of GERD, reflux esophagitis and metaplasia	Collection of oral, esophageal and gastric samples; 16S rRNA sequencing; comparative analysis of the microbiome by disease and treatments (including PPI)	Children with reflux/esophagitis vs children without severe impairment; comparisons between disease stages (GERD vs esophagitis vs metaplasia)	Diversity and composition of the microbiome in the upper GI tract; the degree of ‘microbial similarity’ between compartments; the effect of reflux and PPI on the microbiome
Parashette et al. (26)	Characterization of the esophageal microbiome in healthy children and children with EoE, including reflux-associated forms (esophageal reflux) and PPI-dependent EoE	Esophageal biopsies; 16S rRNA sequencing for esophageal microbiome analysis	Healthy vs RE vs. PPI-REE vs EoE	Diversity and composition of the esophageal microbiome; differences between groups (e.g., *Streptococcus, Haemophilus, Prevotella*); association of eosinophilic inflammation and reflux with microbial changes
Pantazi et al. (27)	Analysis of gut microbiota composition in infants with FGIDs, including GER, and association with clinical and environmental factors	Analysis of intestinal microbiota from fecal samples by selective bacteriological/cultural methods; evaluation of clinical factors (type of diet, mode of birth, etc.)	Infants with FGIDs versus infants without FGIDs	Composition of the intestinal microbiota; degree of dysbiosis; levels of *Lactobacillus, Bifidobacterium, Enterococcus* vs *Enterobacteriaceae*; association of reflux with dysbiosis
Ye et al. (28)	Investigation of changes in gut microbiota and metabolic profile in children with GERD and identification of the microbiota–metabolome relationship in disease pathogenesis	Analysis of fecal microbiota by 16S rRNA + metagenomic sequencing; LC-MS metabolomic analysis of circulating metabolites	Children with GERD vs. Healthy Children	Diversity and composition of the gut microbiota; functional changes (metabolic pathways); differential metabolites (e.g., arachidonic acid, tyrosine, glutathione, caffeine metabolism); Microbiota–metabolome correlations
Zhao et al. (29)	Evaluation of the pharmacokinetic profile of esomeprazole in children with GERD symptoms	Administration of oral esomeprazole in weight-adjusted doses; Measurement of plasma concentrations and pharmacokinetic parameters	No placebo comparator (open-label); Age/dose group comparisons	Pharmacokinetic parameters: Cmax, Tmax, AUC, clearance; inter-individual variability; Safety
Gilger et al. (30)	Evaluation of the safety and tolerability of esomeprazole and the effect on GERD symptoms in children	Administration of esomeprazole 5, 10 or 20 mg/day for 8 weeks	There is no formal placebo comparator; Comparisons between doses and weights ( < 20 kg vs. ≥ 20 kg)	Adverse events, clinical safety, GERD symptomatic scores, symptom relief
Winter et al. (31)	Evaluation of the efficacy and safety of esomeprazole in the treatment of GERD in infants	Esomeprazole dosed according to weight (2.5–10 mg/day) after an initial open-label response phase	Esomeprazole vs placebo (after randomization to respondent patients)	Time until symptoms worsen; symptomatic scores (vomiting, irritability); Safety and adverse events
Omari et al. (32)	Evaluation of the pharmacokinetics and gastric acid suppression effect of esomeprazole in infants with GERD	Oral esomeprazole 0.25 mg/kg vs 1 mg/kg once daily for 1 week	Low dose vs high dose (0.25 mg/kg vs. 1 mg/kg)	Pharmacokinetic parameters (AUC, Cmax), gastric and esophageal pH, number of acid reflux episodes, safety and tolerability
Zhang et al. (33)	Evaluation of the effect of PPI on the oral and intestinal microbiome in children, in longitudinal dynamics	PPI administration for ∼8 weeks; collection of fecal and oropharyngeal samples before and after treatment; microbiome analysis by 16S rRNA sequencing	Each patient serves as their own control (pre-PPI vs post-PPI)	Changes in the fecal and oropharyngeal microbiome; increase of oropharyngeal bacteria in the intestine (e.g., *Streptococcus*); reduction of some beneficial taxa (e.g., *Bifidobacterium*); the degree of oral-intestinal microbial “translocation”
Rajagopala et al. (34)	Evaluation of the effect of PPI on esophageal mucosal gene expression and esophageal microbiome in children	Esophageal biopsies; transcriptomic analysis and microbiome sequencing	Comparison of samples before vs after exposure to PPI (or PPI vs. non-PPI, depending on the subgroup)	Changes in the expression of genes involved in inflammation and epithelial barrier; changes in the esophageal microbiome (bacterial diversity and composition)
Simakachorn et al. (35)	Assessment of changes in the gut microbiome in children after short-term use of PPI	PPI treatment 4–8 weeks; collection of fecal samples before and after treatment; microbiome analysis by 16S rRNA sequencing	Same pre-PPI vs. post-PPI patients	Diversity of the gut microbiome; changes in bacterial composition (phylum-level: *Firmicutes, Bacteroidetes*); possible correlations with infectious adverse symptoms
Francis et al. (36)	Evaluation of the effect of GER and PPI exposure on the gut microbiome in very young infants	Collection of fecal samples; 16S rRNA sequencing of the gut microbiome; Alpha- and beta-diversity analysis	Infants with reflux treated with PPI vs healthy infants without reflux/PPI	Gut microbiome diversity (α and β diversity); changes in bacterial composition (e.g., increase of Firmicutes in the GER+PPI group, dominance of Bifidobacterium in controls); PPI–dysbiosis correlation
Castellani et al. (37)	Evaluation of the effect of PPI on fecal microbiota in infants with gastroesophageal reflux	Administration of PPI (esomeprazole) for several weeks; collection of fecal samples before and after treatment; microbiome analysis by 16S rRNA sequencing	Each patient serves as their own control (pre-PPI vs post-PPI)	Changes in the diversity and composition of the intestinal microbiota; correlations with acid exposure and suppression
Cares et al. (38)	Risk assessment of SIBO in children chronically using PPI	Chronic PPI use ( > 6 months); testing for SIBO by hydrogen/methane breath test (glucose H_2_/CH_4_ breath test)	Children treated with PPI vs children without PPI	SIBO prevalence (based on breath test); association between the use of PPI and the emergence of SIBO
Sieczkowska et al. (39)	Evaluation of SIBO occurrence and persistent symptoms in children treated with PPI for peptic esophagitis	Treatment with omeprazole for 3 months; SIBO testing by hydrogen/methane breath test before and after treatment	Same patients before vs after PPI treatment (pre-post)	SIBO prevalence after PPI; gastrointestinal symptoms (abdominal pain, bloating, belching, flatulence) associated with SIBO
Hegar et al. (40)	Evaluation of the incidence of SIBO in children treated with omeprazole and the effect of probiotics on SIBO prevention	Omeprazole 20 mg/day for 4 weeks + probiotics *(Lactobacillus rhamnosus* R0011 + *Lactobacillus acidophilus* R0052)	PPI + probiotics vs PPI + placebo	Incidence of SIBO (glucose breath test + suggestive symptoms); digestive symptoms (bloating, diarrhea, flatulence); Efficacy of Probiotics
Belei et al. (41)	Evaluation of the Effect of Probiotics (*Lactobacillus reuteri* DSM 17938) on Dysbiosis/SIBO in Children with GERD Treated with PPI	Administration of esomeprazole + probiotics vs esomeprazole + placebo for 12 weeks	PPI + probiotics vs PPI + placebo; plus healthy control group	Prevalence of SIBO (GHBT respiratory test), gastrointestinal symptoms, dysbiosis differences
Dziechciarz et al. (42)	Evaluation of the efficacy of *Lactobacillus rhamnosus* GG (LGG) in the prevention of gastrointestinal and respiratory infections in children with GERD treated with PPI	LGG administration (10^9^ CFU 2 times/day) vs placebo during PPI treatment	LGG + PPI vs placebo + PPI	Incidence of gastrointestinal and respiratory infections; adverse events; Probiotic Safety
Mitre et al. (43)	Evaluation of the association between the use of acid suppressant drugs (PPI/H2RA) and antibiotics in the first 6 months of life and the subsequent development of allergic diseases	Exposure to PPI, H2-blockers, and antibiotics during the first 6 months of life	Children exposed vs. not exposed to acid-suppressant medication/antibiotics	Development of allergic diseases: food allergies, asthma, allergic rhinitis, atopic dermatitis, anaphylaxis, urticaria, allergic conjunctivitis, etc.; Hazard ratios for each category
Chaaban et al. (44)	Evaluation of the association between the use of acid suppressant drugs (PPI/H2RA) and antibiotics in the first year of life and the subsequent risk of food allergies and anaphylaxis	Exposure to PPI, H2-blockers and/or antimicrobials in the first year of life	Exposed vs unexposed children;	Risk of food allergy, anaphylaxis and atopic dermatitis at 2 years; Risk ratios for each exposure category
Lassalle et al. (45)	Assessing the association between PPI use and the risk of severe infections in young children	Exposure to PPI (and other antacid drugs: H2 blockers, antacids); Exposure analysis as a time-dependent variable	Children exposed to PPI vs children not exposed to PPI (within the population cohort)	Incidence of severe hospitalized infections (digestive, respiratory, ENT, urinary, neurological; bacterial and viral); Hazard ratio for risk of infection
Bîrluţiu et al. (46)	Description of a case of Streptococcus agalactiae meningoencephalitis associated with GERD and chronic use of PPI in an infant	Prolonged exposure to PPI in the context of GERD; clinical, microbiological and imaging evaluation	No comparator	Clinical course of severe infection; identification of Streptococcus agalactiae; possible association between PPI, gastric barrier alteration and susceptibility to infections
van der Sande et al. (47)	Assessment of the risk of community pneumonia (CAP) in children treated with gastric acid suppressant drugs (PPI/H2RA)	Exposure to PPI and/or H2-blockers	Children exposed to PPI/H2RA vs. unexposed children	Incidence of community pneumonia (CAP); hazard ratios depending on the type and duration of treatment; The effect of associated respiratory diseases
Freedberg et al. (48)	Evaluation of the association between the use of gastric acid suppressant medication and the risk of CDI in children	Exposure to PPI and/or H2-blockers within the past 8–90 days	Children exposed to acid-suppressants vs unexposed children	CDI risk; odds ratio for CDI depending on the type and timing of exposure to acid suppressants
Stark et al. (49)	Evaluation of the association between the use of antibiotics and stomach acid suppressant medication in the first 2 years of life and the subsequent prescription of psychotropic drugs in childhood	Exposure to PPI, H2-blockers, and/or antibiotics in the first 2 years of life	Children exposed vs. not exposed to acid-suppressants/antibiotics	Subsequent prescriptions of psychotropic drugs (antidepressants, stimulants, antipsychotics, etc.); hazard ratios by type and number of exposures
Wang et al. (50)	Assessing the association between PPI use and the risk of depression and anxiety in children	Initiation of treatment with PPI (omeprazole, esomeprazole, etc.)	PPI users vs non-suitable users by age and propensity score	Incidence of depression and anxiety (clinical diagnosis and/or prescription of antidepressants); hazard ratios depending on the duration of exposure
Gudnadottir et al. (51)	Evaluation of the association between prenatal and early childhood exposure to antibiotics or PPI and the subsequent risk of epilepsy in children	Maternal prenatal exposure and/or early childhood exposure to antibiotics, PPI, and H2-blockers	Exposed vs. non-exposed children to antibiotics/acid-suppressants	Incidence of epilepsy and relative risk associated with early drug exposure
Gudnadottir et al. (52)	Assessment of the association between prenatal and early childhood exposure to PPI and antibiotics and childhood cancer risk	Maternal prenatal exposure and/or exposure in the first years of life to PPI and antibiotics	Children exposed vs. not exposed to PPI/antibiotics	Childhood cancer incidence (all types + subtypes); hazard ratios for exposure risk

16S rRNA, 16S ribosomal ribonucleic acid; AUC, area under the concentration–time curve; CAP, community-acquired pneumonia; CDI, Clostridioides difficile infection; CFU, colony-forming units; EoE, eosinophilic esophagitis; ENT, ear, nose and throat; FGIDs, functional gastrointestinal disorders; GER, gastroesophageal reflux; GERD, gastroesophageal reflux disease; GHBT, glucose hydrogen breath test; GI, gastrointestinal; H_2_RA, histamine-2 receptor antagonist; LC-MS, liquid chromatography–mass spectrometry; LGG, Lactobacillus rhamnosus GG; PPI, proton pump inhibitor; PPI-REE, proton pump inhibitor-responsive esophageal eosinophilia; RE, reflux esophagitis; SIBO, small intestinal bacterial overgrowth; Tmax, time to maximum plasma concentration; Cmax, maximum plasma concentration.

**TABLE 3:** Risk of bias assessment and methodological quality of included studies.

Study	Study design	Risk of bias tool	Score / Overall assessment	Main source of bias
Duvallet et al. (22)	Prospective observational cohort	Newcastle–Ottawa Scale (NOS)	8/9 – Good quality (Low risk of bias)	Small sample size, observational design, residual confounding due to clinical heterogeneity.
Boers et al. (23)	Prospective observational study	Newcastle–Ottawa Scale (NOS)	7/9 – Good quality (Moderate risk of bias)	Small pilot study, GER defined mainly by parental questionnaire, absence of objective reflux diagnosis.
Harris et al. (24)	Cross-sectional study	JBI Critical Appraisal Checklist (Cross-sectional)	7/8 – Moderate-to-high quality (Moderate risk of bias)	Cross-sectional design, mixed pediatric/adult population, potential treatment-related confounding.
Luu et al. (25)	Prospective observational study	Newcastle–Ottawa Scale (NOS)	8/9 – Good quality (Low risk of bias)	Small prospective cohort, treatment heterogeneity, residual confounding.
Parashette et al. (26)	Cross-sectional study	JBI Critical Appraisal Checklist (Cross-sectional)	7/8 – Moderate-to-high quality (Moderate risk of bias)	Small sample, single time-point sampling, influence of PPI exposure.
Pantazi et al. (27)	Longitudinal prospective cohort study	Newcastle–Ottawa Scale (NOS)	8/9 – Good quality (Low risk of bias)	Culture-based microbiota analysis, residual confounding (diet, antibiotics, birth mode).
Ye et al. (28)	Case–control study	Newcastle–Ottawa Scale (Case–Control)	8/9 – Good quality (Low risk of bias)	Cross-sectional comparison, relatively small sample, residual confounding.
Zhao et al. (29)	Randomized open-label pharmacokinetic trial	Cochrane RoB 2	Some concerns	Open-label design despite objective pharmacokinetic outcomes.
Gilger et al. (30)	Randomized controlled multicenter trial	Cochrane RoB 2	Some concerns	No placebo comparator, short follow-up, safety-oriented design.
Winter et al. (31)	Randomized double-blind placebo-controlled trial	Cochrane RoB 2	Some concerns	Enriched design after open-label run-in phase, subjective symptom assessment in infants.
Omari et al. (32)	Randomized single-blind trial	Cochrane RoB 2	Some concerns	Single-blind design, no placebo, short follow-up.
Zhang et al. (33)	Longitudinal prospective observational study	Newcastle–Ottawa Scale (NOS)	8/9 – Good quality (Low risk of bias)	No external control group, residual confounding (diet, antibiotics, disease severity).
Rajagopala et al. (34)	Prospective observational cohort study	Newcastle–Ottawa Scale (NOS)	8/9 – Good quality (Low risk of bias)	Small exploratory sample, lack of randomization, residual confounding.
Simakachorn et al. (35)	Prospective observational before–after study (non-randomized interventional)	JBI Critical Appraisal Checklist for Quasi-Experimental Studies	7/9 – Moderate quality (Moderate risk of bias)	Small sample size, absence of an external control group, short follow-up period, potential confounding from age, diet, antibiotic exposure and underlying indication for PPI therapy; inability to distinguish microbiome changes related to PPI exposure from natural microbiome maturation.
Francis et al. (36)	Prospective observational cohort study	Newcastle–Ottawa Scale (NOS) – Cohort Studies	7/9 – Moderate quality (Moderate risk of bias)	Small sample size, lack of randomization, residual confounding related to age, feeding type (breastfeeding/formula), prematurity, maternal factors and indication for PPI therapy; difficulty separating the independent effects of GER and PPI exposure on microbiome changes.
Castellani et al. (37)	Prospective before–after quasi-experimental study (non-randomized intervention)	JBI Critical Appraisal Checklist for Quasi-Experimental Studies	7/9 – Moderate quality (Moderate risk of bias)	Small sample size, absence of an external control group, short follow-up period, potential confounding from age-related maturation of the infant microbiome, diet, feeding pattern and underlying indication for PPI therapy; inability to separate microbiome changes caused by PPI exposure from natural microbiome development.
Cares et al. (38)	Case–control study	Newcastle–Ottawa Scale (Case–Control)	8/9 – Good quality (Low risk of bias)	Small sample size, limited adjustment for confounders, and potential misclassification of SIBO diagnosis due to use of hydrogen breath test rather than jejunal aspirate.
Sieczkowska et al. (39)	Prospective before–after quasi-experimental study (non-randomized intervention)	JBI Critical Appraisal Checklist for Quasi-Experimental Studies	7/9 – Moderate quality (Moderate risk of bias)	Small sample size, absence of an external control group, short follow-up period, SIBO diagnosis based on hydrogen breath test rather than jejunal aspirate (gold standard), potential confounding from underlying disease severity, diet, antibiotic exposure and PPI indication; inability to establish causality between PPI exposure and SIBO persistence.
Hegar et al. (40)	Randomized double-blind placebo-controlled trial	Cochrane RoB 2	Low risk of bias	Well-conducted RCT; only limitation is relatively small sample size.
Belei et al. (41)	Randomized double-blind controlled trial	Cochrane RoB 2	Some concerns	No non-PPI arm, microbiota assessed indirectly through GHBT, relatively small sample.
Dziechciarz et al. (42)	Randomized double-blind placebo-controlled trial	Cochrane RoB 2	Some concerns	Underpowered study due to difficult recruitment, short follow-up, parent-reported outcomes.
Mitre et al. (43)	Retrospective population-based cohort study	Newcastle–Ottawa Scale (NOS)	9/9 – Good quality (Low risk of bias)	Large administrative database, objective exposure assessment; residual confounding by indication, lack of microbiome, diet, breastfeeding and family history data.
Chaaban et al. (44)	Retrospective population-based cohort study	Newcastle–Ottawa Scale (NOS)	9/9 – Good quality (Low risk of bias)	Large database-based cohort; potential residual confounding, ICD-code misclassification, lack of microbiome and lifestyle data.
Lassalle et al. (45)	Retrospective nationwide cohort study	Newcastle–Ottawa Scale (NOS)	9/9 – Good quality (Low risk of bias)	Large population cohort with time-dependent exposure analysis; residual confounding due to GERD severity, underlying disease, and incomplete clinical information.
Bîrluţiu et al. (46)	Case report	JBI Critical Appraisal Checklist (Case Report)	6/8 – Moderate quality (High risk of bias)	Single case without comparator, inability to establish causality, multiple potential predisposing factors.
van der Sande et al. (47)	Retrospective population-based cohort study	Newcastle–Ottawa Scale (NOS)	9/9 – Good quality (Low risk of bias)	Large registry-based cohort with objective exposure/outcome assessment; residual confounding by indication, absence of microbiome and lifestyle information.
Freedberg et al. (48)	Nested case–control study	Newcastle–Ottawa Scale (Case–Control)	9/9 – Good quality (Low risk of bias)	Population-based design with objective exposure assessment; possible residual confounding, administrative coding limitations, lack of microbiome data.
Stark et al. (49)	Retrospective population-based cohort study	Newcastle–Ottawa Scale (NOS)	9/9 – Good quality (Low risk of bias)	Large longitudinal cohort; psychotropic prescriptions used as proxy for neuropsychiatric disorders, residual confounding by underlying illness and socioeconomic/familial factors.
Wang et al. (50)	Nationwide retrospective cohort study	Newcastle–Ottawa Scale (NOS)	9/9 – Good quality (Low risk of bias)	Robust registry-based design and propensity score adjustment; residual confounding related to psychosocial factors, genetic predisposition, and disease severity.
Gudnadottir et al. (51)	Nationwide retrospective population-based cohort study	Newcastle–Ottawa Scale (NOS)	9/9 – Good quality (Low risk of bias)	Large national cohort with validated registers; potential confounding by maternal/child health status, genetic susceptibility, and lack of microbiome data.
Gudnadottir et al. (52)	Nationwide retrospective population-based cohort study	Newcastle–Ottawa Scale (NOS)	9/9 – Good quality (Low risk of bias)	Large registry-based cohort; rare outcome events, residual confounding, absence of immunological, genetic and microbiome-related data.

GHBT, glucose hydrogen breath test; GER, gastroesophageal reflux; GERD, gastroesophageal reflux disease; JBI, Joanna Briggs Institute; NOS, Newcastle–Ottawa Scale; PPI, proton pump inhibitor; RCT, randomized controlled trial; RoB 2, Risk of Bias 2; SIBO, small intestinal bacterial overgrowth.

### 3.1 Disease-associated microbiota alterations in pediatric GERD

Analyzing the specialized literature, in a systematic manner, we observe a low density of publications regarding the modification of the microbiome in GERD, specifically for the pediatric population. At the same time, the existing research is heterogeneous, both in terms of the characteristics of the integrated population, outcomes, the bacterial taxa studied, the technique of their investigation, and, especially, the microbial sites of interest. However, analyzing the existing data, we can roughly conclude that the authors convergently suggest that GER, aspiration and esophageal inflammation are associated with changes in the microbiome at the level of several anatomical sites investigated, including the oropharynx, nasopharynx, middle ear, lung, esophagus, stomach and intestine. The described changes include alterations in bacterial diversity, changes in microbial composition and increased similarity between the microbiota of different compartments of the digestive and respiratory tract, suggesting a possible microbial transfer facilitated by reflux or aspiration.

At the aerodigestive tract level, Duvallet et al. noted that all three aerodigestive compartments share common predominant taxa, notably the genera *Streptococcus, Prevotella, Haemophilus, Veillonella* and *Neisseria*, but at the operational taxonomic unit (OTU) level the microbial communities remain distinct. The oropharyngeal microbiome was found to be relatively stable between individuals, whereas the lung and gastric microbiomes showed marked interindividual variability, being predominantly influenced by individual patient characteristics rather than anatomical site. In the intraindividual analysis, the lowest microbiological similarity was observed between the oropharynx and lung (median JSD = 0.90), suggesting that, under physiological conditions, airway protective mechanisms limit the transfer of oropharyngeal flora to the lower respiratory tract. Surprisingly, stomach–lung similarity was comparable to stomach–oropharynx similarity (median JSD = 0.56), suggesting the existence of complex interactions between aerodigestive compartments, independent of aspiration. However, the central finding of the study was that patients with dysphagia and aspiration showed a significant convergence between the lung and oropharyngeal microbiomes, without a corresponding increase in gastric–lung similarity. Regarding GER, subgroup analysis of patients revealed moderate correlations between reflux severity and stomach–lung microbiological similarity, especially for proximal reflux and full column reflux episodes (e.g., proximal reflux: ρ = -0.55, *p* = 0.002), suggesting that reflux may contribute to some extent to the upward microbial transfer. However, the magnitude of these associations was modest compared to the oropharynx–lung relationship, and the lack of a significant increase in common stomach–lung OTUs in aspirators argues against a dominant role of gastric reflux in the pathogenesis of pulmonary complications (22). Boers et al. also investigated the possible implications of GER on the nasopharyngeal and middle ear microbiota in children with otitis media (OM). The results showed that there were no significant differences between the microbiota of reflux-prone and non-reflux-prone children, both in the nasopharynx and middle ear. These findings suggest that GER does not directly influence the composition of the microbiota associated with OM. The most frequently identified bacteria in middle ear fluid were *Alloiococcus* spp. and *Turicella* spp., but these were not detected in nasopharyngeal swabs, suggesting that they are not classical primary pathogens of OM. In contrast, classical bacteria implicated in OM, such as *Haemophilus* spp., *Streptococcus* spp. and *Moraxella* spp., were identified in middle ear samples, and *Haemophilus* and *Streptococcus* were detected in the ear only when they simultaneously colonized the nasopharynx. The study did not identify gastrointestinal bacteria such as *Helicobacter pylori* in the middle ear and did not demonstrate translocation of gastric microbiota to the ear in children with reflux. The authors conclude that GER does not appear to significantly alter the nasopharyngeal or middle ear microbiota in children with OM, but emphasize the importance of further investigation of the role of *Alloiococcus* spp. and *Turicella* spp. in the pathogenesis of pediatric OM (23).

The esophageal microbiota, a segment primarily affected by GERD flares, was comprehensively analyzed by Harris et al., comparing changes between children and adults diagnosed with active eosinophilic esophagitis (EoE), EoE in remission, GERD, and controls with normal esophageal mucosa. The authors hypothesized that eosinophilic inflammation and changes in the epithelial barrier in EoE may alter the composition of the local microbiota, and that GER and PPI treatment may further modulate these bacterial communities. Quantitative analysis by qPCR demonstrated that both EoE and GERD are associated with a significantly increased bacterial load compared to normal esophagus, independent of treatment or the degree of histological activity of EoE, suggesting that esophageal inflammation may be accompanied by global bacterial expansion. However, alpha diversity did not differ significantly between groups, indicating that the inflammatory process mainly modifies bacterial abundance and not global taxonomic richness. At the phylogenetic level, the esophageal microbiome was dominated by four major phyla - *Firmicutes, Proteobacteria, Bacteroidetes*, and *Fusobacteria*—in both EoE and GERD and controls, but relevant changes were observed at the genus level. Regarding GERD, the study identified distinct changes associated with both the disease and PPI therapy. Patients with untreated GERD had an increase in *Firmicutes* and a decrease in *Proteobacteria* compared with normal subjects, but PPI treatment resulted in a marked expansion of *Proteobacteria*, especially the genus *Aggregatibacter*, concomitant with a relative reduction of *Streptococcus* from 47 to 21%. This observation is particularly important because it suggests that PPI were associated with significant alterations in the esophageal microbial ecology, likely by alkalinizing gastric reflux and altering the selective pressure exerted by acid on the microbiota. The authors also discuss the possibility of a direct effect of PPI on bacterial ATPase-type proton pumps found in *Streptococcus* spp., which could explain the reduction of this genus in treated GERD. Interestingly, these PPI-induced changes were not observed to the same extent in subjects with EoE or normal controls, suggesting that the effect of antacid therapy on the microbiome depends on the local inflammatory and pathophysiological context (24).

Similarly, analyzing the discrepancy between the upper gastroesophageal microbiome in children with GER and esophageal metaplasia, Luu et al. reiterated that the pediatric esophageal microbiota represents a distinct ecosystem from the oral and gastric microbiota, with a lower bacterial diversity, although the majority of esophageal bacteria originate from the oral cavity. GER was associated with a homogenization of the oral, esophageal, and gastric microbiota, suggesting that repeated exposure to reflux alters the structure of bacterial communities in the upper digestive tract. The most important observation of the study was the association of esophageal metaplasia with increased prevalence and abundance of the genus *Prevotella*, especially the taxon similar to *Prevotella melaninogenica* (*P. melaninogenica*). In parallel, the authors observed a reduction in *Streptococcus* species and a progressive increase in *Prevotella* from normal esophagus to GERD, EoE and metaplasia. Genomic analysis revealed that *P. melaninogenica* strains present in metaplasia contained distinct genetic features, including membrane proteins involved in bacterial adaptation and possibly in inflammatory mechanisms. The study also highlighted the impact of PPI use on the gastric microbiota. Children on PPI treatment showed increased gastric bacterial diversity and a greater contribution of oral bacteria to the stomach, changes associated with increased levels of proinflammatory cytokines. The authors suggest that reduced gastric acidity may allow oral bacteria to survive gastric transit and colonize the stomach, potentially contributing to local inflammation (25). Complementing the findings, Parashette et al. investigated the esophageal microbiome in healthy children and children with esophageal eosinophilia, including reflux esophagitis (RE), proton pump inhibitor-responsive esophageal eosinophilia (PPI-REE), and EoE. The most prevalent bacterial phyla identified were *Actinobacteria, Bacteroidetes, Firmicutes, Fusobacteria*, and *Proteobacteria*. Although the healthy group of children showed higher microbial diversity and richness compared to the pathological groups, the differences were not statistically significant. Beta-diversity analysis showed that the genera *Streptococcus, Rahnella*, and *Leptotrichia* explained approximately 30% of the variation in microbiota composition, and *Microbacterium, Prevotella*, and *Vibrio* contributed additionally to the differences observed between groups. Particularly in the case of RE, the authors observed changes in the composition of the esophageal microbiome compared to healthy children. The order *Vibrionales* was predominant in healthy children and those with EoE but reduced in the reflux and PPI-REE groups. Also, certain bacterial classes and orders, such as *Rhodospirillales*, showed significant differences between groups. The study supports previous observations that the esophageal microbiome is altered in GERD, but the authors emphasize that it is not yet clear whether the changes in the microbiota are the cause or consequence of esophageal inflammation (26).

Regarding GER in infants, the study by Pantazi et al. revealed significant changes in the intestinal microbiota compared to infants without functional gastrointestinal disorders. Infants diagnosed with GER showed low levels of beneficial bacteria, especially *Lactobacillus* spp. and *Bacteroides* spp., along with an increase in opportunistic and proteolytic bacteria such as *Enterococcus* spp., *Escherichia coli* and *Klebsiella* spp. These changes were associated with an intestinal dysbiosis profile of intermediate severity, as suggested by the flora index values. The authors emphasize that the imbalance between protective flora and proteolytic bacteria may influence gastrointestinal function and may contribute to reflux symptoms during the first year of life. The study also showed that factors such as cesarean section birth and artificial feeding favor the onset of intestinal dysbiosis and increase the risk of developing functional gastrointestinal disorders, including GER (27). Ye et al. complement the findings in a larger population of children, noting the existence of significant changes in the intestinal microbiota in GERD children. Compared to the healthy group, children with GERD showed reduced intestinal microbial diversity and a significant change in bacterial composition, characterized by an increase in the abundance of species from the phylum *Proteobacteria* and *Bacteroidetes* and a decrease in *Firmicutes* and *Actinobacteria*. At the genus and species level, bacteria such as *Bacteroides stercoris, Bacteroides vulgatus, Alistipes putredinis, Escherichia-Shigella, Klebsiella* and *Haemophilus*, associated with inflammatory processes and the production of lipopolysaccharides, predominated. At the same time, bacteria considered beneficial, producing short-chain fatty acids (SCFAs), such as *Bifidobacterium, Faecalibacterium, Blautia* and *Lachnospira*, were significantly reduced. Functional analysis of the microbiota revealed alterations in metabolic pathways involved in energy, amino acid, vitamin, carbohydrate and lipid metabolism. Metabolomic analysis identified 288 differential metabolites between groups, with significant changes in the metabolism of arachidonic acid, tyrosine, glutathione and caffeine. Increased levels of proinflammatory metabolites, including leukotriene B4, suggest the involvement of the systemic inflammatory response in the pathogenesis of GERD (28).

### 3.2 PPI-associated microbiota alterations

Therefore, without directly targeting the microbial changes that accompany GERD, but rather the acute symptomatology of patients, the widespread use of PPI as the main drug ally has become routine in medical practice. With this, interest in its safety, tolerability and main side effects due to exposure has also increased. In this note, analyzing the pediatric population with GERD, Zhao et al. evaluated the pharmacokinetic properties and tolerability of esomeprazole in children, in a randomized, open-label study. The results showed that the pharmacokinetic properties of esomeprazole are both dose- and age-dependent. In children aged 1–5 years, the administration of a 10 mg dose resulted in AUC and Cmax values more than twice as high compared to the 5 mg dose. In the 6–11 years old group, the systemic exposure after the 20 mg dose was approximately double that observed with the 10 mg dose. Also, the oral clearance adjusted for body weight was approximately 50% higher in younger children compared to older children, suggesting a more rapid metabolism of esomeprazole in the first years of life. The time to reach maximum plasma concentration was less than 2 h in all groups and the plasma half-life was less than 1 h. In addition, the systemic exposure achieved at the doses used in children was comparable to that observed in adults treated with 20 mg esomeprazole. Only a few mild adverse reactions were reported, such as skin excoriations, change in stool color and one skin laceration, none of which were considered related to treatment (29). Gilger et al. also conducted a multicenter, randomized, double-blind, 8-week study. Participants were divided according to weight and received daily doses of esomeprazole of 5 mg, 10 mg, or 20 mg. The study findings showed that esomeprazole was generally well tolerated, with only 9.3% of children experiencing adverse reactions considered to be related to treatment, the most common being diarrhea, headache, and drowsiness. No severe adverse reactions directly attributable to the medication were identified. In addition, treatment resulted in a significant reduction in symptoms of GERD, including heartburn, acid regurgitation, and epigastric pain, with most patients showing improvement in clinical scores by the end of the study (30). In line with these findings, Winter et al. and Omari et al. studied the efficacy and safety of PPI in infants. Winter et al. conducted a multicenter, randomized, double-blind, placebo-controlled study conducted in two phases: an initial 2-week open-label phase, followed by a 4-week double-blind phase. In the open-label phase, all infants received weight-adjusted doses of esomeprazole (2.5–10 mg/day), and those who showed improvement in symptoms were subsequently randomized to continue treatment with esomeprazole or placebo. Results showed that 82.7% of infants showed improvement in symptoms during the open-label phase, but in the placebo-controlled phase the difference between the esomeprazole and placebo groups was not statistically significant in terms of symptom worsening (HR 0.69; *p* = 0.28). *Post-hoc* analyses suggested a possible benefit of esomeprazole in infants with symptomatic GERD not confirmed by diagnostic investigations, in whom the time to symptom worsening was significantly longer compared to placebo (HR 0.24; *p* = 0.01). In terms of safety, esomeprazole was well tolerated, with the most common adverse reactions being upper respiratory tract infections, fever, rhinitis and diarrhea, with no serious adverse events considered related to treatment. In the study by Omari et al., the results showed a dose-dependent relationship in terms of both systemic exposure to the drug and the gastric acid suppression effect. AUC0–t values demonstrated a significant increase in esomeprazole exposure with increasing dose. After treatment, the percentage of time with gastric pH > 4 increased significantly, concomitantly with a reduction in esophageal acid exposure and the number of episodes of prolonged acid reflux. The plasma half-life of esomeprazole was approximately 0.8–1 h, and interindividual variability in pharmacokinetic parameters was more pronounced in infants under 12 months of age, possibly due to incomplete maturation of the CYP2C19 and CYP3A4 enzymes involved in drug metabolism (31, 32). Thus, data in young children are contradictory, supporting the good tolerance and effectiveness of esomeprazole in acid suppression in infants with GERD, but still emphasizing the need to outline precise diagnostic and therapeutic initiation readings.

Integrating these data into our research theme, we were further interested in discussing the possible link between PPI administration in children and microbial dysbiosis. In this direction, Zhang et al. longitudinally evaluated changes in the intestinal and oropharyngeal microbiome in children treated with PPI. The prospective study included children and adolescents from whom stool and oropharyngeal exudate samples were collected before the initiation of therapy and after 8 weeks of PPI treatment. Microbiome analysis by 16S rRNA sequencing revealed that, although there were no significant changes in the overall bacterial diversity (alpha and beta-diversity), PPI use was associated with important changes in the level of certain bacterial taxa. Thus, after treatment, an increase in the abundance of the genus *Streptococcus* in the intestinal microbiome and a decrease in *Bifidobacterium*, *Peptostreptococcaceae* and *Turicibacter* were observed. The researchers also demonstrated an increase in bacteria of oropharyngeal origin in the intestine after PPI administration, a phenomenon that was more pronounced in young children. The authors suggest that reduced gastric acidity may facilitate the survival and migration of oral microorganisms to the distal gastrointestinal tract, which may explain the association between PPI use and increased risk of gastrointestinal and respiratory infections in children (33). Rajagopala et al. investigated the effects of PPI exposure on the esophageal mucosal transcriptome and active microbiome in children with endoscopically and histologically normal esophagus. The results of metatranscriptomic analysis revealed the up-expression of 27 genes in PPI-treated patients, including genes involved in homeostasis and protection of the esophageal epithelium, such as MUC2, MUC3A, DMBT1, and PIGR, suggesting the activation of mucin secretion and epithelial differentiation mechanisms. Deconvolutional analysis of cellular composition showed that these changes were not determined by differences in the proportion of cell types, but by direct effects of PPI on gene expression. Regarding the microbiome, the PPI-exposed group showed an increased abundance of *Streptococcus* spp. and *Prevotella* spp., without significant changes in alpha or beta diversity. The authors conclude that PPI use was associated with alterations in the molecular and microbiological profile of the esophageal mucosa even in the absence of obvious esophageal pathology (34).

Similar findings were reported by Simakachorn et al., who evaluated changes in the gut microbiota in a prospective observational study of children treated with PPI for 4–8 weeks. Analysis of the fecal microbiota showed that, before and after treatment, the gut microbiota was dominated by bacteria belonging to the phylum Bacteroidetes, with no significant changes in the total number of bacterial species, alpha or beta diversity, or overall microbiota composition. However, the authors observed a trend toward an increase in the proportion of bacteria from the phylum Firmicutes in certain subgroups of children, particularly those living in urban/metropolitan areas and in boys. Also, in children who experienced minor infectious adverse effects during treatment, such as diarrhea or upper respiratory infections, a nonsignificant increase in Firmicutes was noted after PPI use. The authors conclude that short-term PPI treatment was not associated with major changes in the pediatric intestinal microbiota, but may influence certain bacterial groups depending on individual and environmental characteristics (35). In very young infants diagnosed with GER, the results obtained by Francis et al. note significant differences between the PPI-exposed group and those in the control group in terms of both alpha and beta diversity of the intestinal microbiota. Infants with reflux treated with PPI showed greater microbial diversity and an increased abundance of several bacterial taxa, especially from the phylum Firmicutes. In contrast, the microbiota of the control group was dominated by Bifidobacterium, bacteria commonly associated with natural feeding and healthy development of the infant microbiota. The authors observed that the duration of PPI exposure was associated with differences in microbiota composition of the microbiota. Longer exposure ( > 30 days) was associated with an increase in bacterial genera such as Flavonifractor, Blautia, Ruminococcaceae, Anaerostipes, and Lachnospiraceae. Some of these bacteria are involved in carbohydrate metabolism and the production of SCFAs, but other studies have also associated them with inflammatory processes or metabolic disorders. The study highlights that infants treated with PPI showed an accelerated maturation of the intestinal microbiota compared to healthy infants. Although increased microbial diversity is often considered favorable in adults, the authors point out that premature maturation of the microbiota in the first months of life could potentially disrupt the normal development of the immune system and intestinal homeostasis. At the same time, the researchers mention the existence of important confounding factors between the groups, such as the higher proportion of artificial feeding, cesarean section births and prematurity in the reflux group. However, after statistical adjustment for age and exclusive breastfeeding, PPI exposure and duration of treatment remained factors significantly associated with changes in the microbiota (36).

These results are in agreement with earlier data presented by Castellani et al. which also targeted infants diagnosed with GERD. The results obtained by collecting and analyzing fecal samples from three different time points: before treatment (“before PPI”), 4 weeks after starting therapy (“on PPI”) and 4 weeks after stopping treatment (“after PPI”) showed that PPI administration was not associated with significant changes in α and β diversity between the pretreatment period and the period in which infants received treatment. However, at the taxonomic level, some point changes in relative bacterial abundance were identified: a decrease in the genera *Lactobacillus* and *Stenotrophomonas* and an increase in the genus *Haemophilus* were observed during PPI therapy. Also, although *Streptococcus* showed a trend of increase, the changes in bacteria commonly associated with *Clostridioides difficile* infection in adults did not reach statistical significance. An important aspect of the study was the fact that the most obvious changes in the microbiome occurred after stopping PPI therapy. At this stage, a significant increase in both α and β diversity was observed, along with changes in the relative abundance of the main bacterial phyla, particularly *Firmicutes, Bacteroidetes*, and *Proteobacteria*. The authors concluded that these changes are more likely related to the normal physiological process of maturation of the gut microbiome, correlated with infant age and the introduction of solid foods, than to a direct effect of PPI (37).

Other authors, such as Cares et al. and Sieczkowska et al. draw attention to the risk of small intestinal bacterial overgrowth (SIBO) associated with chronic use of PPI in children. In the prospective cohort study by Cares et al., although the incidence of SIBO was higher in the exposed group (8.9% vs. 3.7%), the difference was not statistically significant, and the relative risk (RR = 2.4) had a wide confidence interval, suggesting a possible but uncertain association. In contrast, the study by Sieczkowska et al., on 40 children with peptic esophagitis treated with omeprazole revealed a significant increase in SIBO after treatment (22.5%, *p* = 0.011). In addition, the presence of SIBO was associated with more severe and persistent gastrointestinal symptoms (abdominal pain, bloating, flatulence), while patients without SIBO showed improvement of symptoms under PPI. Overall, the two studies suggest that PPI use in children may be associated with changes in gut flora and a possible risk of SIBO, but the level of evidence differs, being inconclusive in the first study and clinically significant in the second, which highlights the need for larger and better controlled research (38, 39).

Turning our attention to this issue, we identified the randomized, double-blind, placebo-controlled clinical trial conducted by Hegar et al., focused on both the assessment of the incidence of SIBO in children treated with omeprazole and the effect of probiotics (*Lactobacillus rhamnosus* R0011 and *Lactobacillus acidophilus* R0052) in preventing this condition. The results showed that approximately 30% of the children had a positive breath test after 1 month of PPI treatment, and 62% of them also presented compatible clinical symptoms. No significant differences were observed between the probiotic and placebo groups in terms of the incidence of SIBO (33% vs 26.5%; *p* = 0.13), suggesting that the probiotics used did not have a protective effect (40). However, Belei et al. evaluated whether the administration of probiotics (*Lactobacillus reuteri* DSM 17938) together with PPI (esomeprazole, for 12 weeks) can reduce the risk of SIBO in children with GERD, compared to a placebo group and a healthy control group. After 12 weeks of treatment, the prevalence of SIBO was significantly lower in the group that received probiotics (6.2%) compared to the placebo group (56.2%), and the values were similar to those of the healthy control group (5%). In addition, children in the placebo group more frequently presented digestive symptoms associated with SIBO, while in the probiotic group the incidence of symptoms was much reduced. The authors conclude that the administration of probiotics was associated with a significantly lower prevalence of dysbiosis/SIBO in children treated with PPI and may have a protective effect (41).

Regarding the risk of developing gastrointestinal and respiratory infections in children diagnosed with GERD and treated with PPI, Dziechciarz et al. concluded the absence of a protective effect of concomitant administration of probiotics (*Lactobacillus rhamnosus* GG). However, the authors acknowledge the premature interruption of the randomized, double-blind, placebo-controlled trial, after only 12 weeks of follow-up after the completion of PPI treatment (4–6 weeks), due to slow recruitment, which may limit the statistical power of the results and the need for further studies (42).

### 3.3 Clinical outcomes potentially associated with PPI-associated dysbiosis

Deepening the effects of PPI use at a systemic level, we find multiple attestations in the specialized literature, possibly referring to an increased risk of atopy, infections, neuropsychiatric manifestations or even the onset of oncological diseases. In this note, the retrospective cohort study conducted by Mitrea et al. evaluated the association between exposure in the first 6 months of life to PPI, H2 antagonists (H2RA) and antibiotics and the subsequent risk of allergic diseases in childhood. The results showed that the use of these drugs in the neonatal and infant period is associated with a significantly increased risk of developing several allergic pathologies, including food allergies, asthma, allergic rhinitis, atopic dermatitis, urticaria and anaphylaxis. The association was stronger for antisecretory drugs (H2RA and especially PPI), which showed consistent increases in the risk of food allergies (with an adjusted hazard ratio of up to ∼2.6 for PPI), as well as a dose-dependent relationship and duration of exposure. The pathophysiological interpretation of the study is that early exposure to drugs that alter the gut microbiome and protein digestion may influence immunological maturation, favoring dysbiosis and increasing susceptibility to allergic sensitization. PPI may amplify this risk both by modifying the gut flora and by reducing the degradation of food proteins, facilitating antigen exposure and IgE-mediated immune responses (43). Subsequent research by Chaaban et al. using the US TriNetX database reinforces this evidence, reiterating that both PPI and H2RAs were associated with a significant increase in the risk of food allergy and anaphylaxis, with stronger effects for PPI for food allergy (RR ≈ 5.33) and for H2RAs for anaphylaxis (RR ≈ 4.48). Antimicrobial exposure was also associated with an increased risk of allergies, including anaphylaxis and food allergy, and the risk increased with the number of prescriptions, suggesting a dose-dependent effect. In the subanalysis of patients with GERD, these associations remained largely valid for food allergy, although the results for anaphylaxis and atopic dermatitis were more variable (44).

Regarding the infectious risk associated with PPI use, Lassalle et al. retrospectively analyzed the cohort of children who received PPI taken from the French Mother-Child EPI-MERES registry, noting that exposure to PPI was associated with a significant increase in the overall risk of severe infections (aHR 1.34; 95% CI 1.32–1.36). Increased risks were observed for multiple sites of infection: digestive tract (aHR 1.52), ENT sphere (aHR 1.47), lower respiratory tract (aHR 1.22), urinary tract (aHR 1.20) and nervous system (aHR 1.31). Also, both bacterial (aHR 1.56) and viral (aHR 1.30) infections were more frequent in children exposed to PPI. The risk remained increased even after discontinuation of treatment, although it progressively decreased over time. The authors discuss several possible biological mechanisms that could explain this association. Reduced gastric acidity favors the survival of ingested pathogens and alters the gastrointestinal microbiota, which may facilitate the development of enteric infections. In addition, PPI may influence immune function, including neutrophil activity, and may indirectly contribute to respiratory infections through mechanisms such as microaspiration or alteration of the gut-lung axis. Because the infant’s microbiota is still developing, PPI-induced disruptions during this period may have important consequences for susceptibility to infections (45). These findings are reinforced by Bîrluţiu et al. who present a clinical case that highlights the possible severe adverse effects associated with chronic PPI use in infants. The study describes the case of a 9-month-old child diagnosed with meningoencephalitis due to *Streptococcus agalactiae* (Group B Streptococcus), in the context of gastroesophageal reflux disease (GERD) treated for a long time with esomeprazole (0.5 mg/kg/day for approximately 4 months). The authors emphasize that *Streptococcus agalactiae* is a common cause of neonatal and infantile meningitis in the first 90 days of life, but the occurrence of GBS meningitis in older children is rare. In this context, the clinical case is considered relevant because it suggests a possible association between chronic PPI use and increased susceptibility to severe invasive infections. The possible mechanisms by which long-term PPI use could contribute to the occurrence of severe infections are reiterated, in the presented case, the observed leukopenia being considered a possible factor favoring bacteremia and crossing the blood-brain barrier by the pathogen. The authors point out that the immunological effects of antisecretory therapy may persist even after treatment is discontinued (46).

At the same time, authors such as Van der Sande et al. and Freedberg et al. analyze the possible relationship between the use of gastric acid suppressants and the risk of community-acquired pneumonia, respectively of *Clostridioides difficile* infection. The results showed that current PPI use was associated with an approximately two-fold increase in the risk of community-acquired pneumonia (aHR 2.05; 95% CI 1.90–2.22), and H2RA use with a similar risk (aHR 1.80; 95% CI 1.67–1.94). The risk increased progressively with the duration of treatment, being highest with prolonged use ≥ 211 days (aHR 2.34 for PPI and 2.63 for H2RAs) and more pronounced in those with respiratory comorbidities such as asthma or a history of pneumonia. The risk also remained elevated up to 7 months after discontinuation of therapy. Regarding *Clostridioides difficile* infection, it is noted that the use of PPI, with or without associated H2RA, was correlated with a marked increase in risk (OR 21.5; 95% CI 4.71–98.6), while the exclusive use of H2RA had a lower and statistically insignificant association (OR 2.64; 95% CI 0.93–10.4). The risk of *Clostridioides difficile* was also dependent on the proximity of exposure: children who had used antisecretory medication in the last 8–90 days had the highest risk of infection (OR 8.02; 95% CI 3.38–19.0), compared to those not exposed (47, 48).

Furthermore, authors such as Stark et al. retrospectively investigated the association between the administration of antibiotics and gastric antisecretory medication in the first 2 years of life and the risk of later prescription of psychotropic medications in childhood. The results demonstrated that early exposure to gastric antisecretory medication and antibiotics was significantly associated with an increased risk of prescription of psychotropic treatments in childhood. Children exposed to H2RAs had an adjusted hazard ratio (aHR) of 1.79 (95% CI: 1.63–1.96), those exposed to PPI an aHR of 1.47 (95% CI: 1.26–1.71), and those exposed to antibiotics an aHR of 1.71 (95% CI: 1.59–1.84). The association was observed for several categories of psychotropics, including stimulants, antidepressants, and antipsychotics. A dose-dependent effect was also highlighted. The risk of prescribing psychotropic medications increased proportionally with the duration of antibiotic and H2RA exposure, as well as the number of antibiotic classes administered. For example, children who received antibiotics for more than 31 days had an aHR of 2.04 (95% CI: 1.88–2.21), compared with those treated for 1–30 days (aHR 1.42). In addition, the administration of four or more antibiotic classes was associated with an even higher risk of subsequent prescription of psychotropic medication (aHR 2.36; 95% CI: 2.13–2.61). Children exposed to multiple classes of drugs that alter the gut microbiota (PPI, H2RAs, and antibiotics) also had a progressively increased risk, reaching an aHR of 4.47 (95% CI: 3.68–5.43) for those exposed to all three drug categories. However, the study cannot demonstrate a direct causal relationship, and the results must be interpreted taking into account possible confounding factors, such as early infections, comorbidities, or socioeconomic factors (49).

Other reports identified were by Wang et al., who investigated the association between PPI use and the risk of depression and anxiety in children and adolescents. The results showed that initiation of PPI treatment was associated with a significantly increased risk of depression and anxiety (HR = 2.61; 95% CI: 2.32–2.94). The risk was increased from the first 30 days of treatment and remained persistent throughout the follow-up period, increasing with duration of use (50). Gudnadottir et al. also investigated the association between prenatal and first 2 years of life exposure to antibiotics, PPI, and H2 receptor antagonists and the risk of developing epilepsy in children. The results showed that prenatal exposure to antibiotics and PPI was associated with an increased risk of epilepsy in children. The risk was more pronounced for PPI, and for antibiotics a dose-dependent relationship was observed, with the risk increasing with the number of prescriptions administered during pregnancy. Regarding early childhood exposure, the administration of antibiotics, PPI, and H2RAs before the age of two was associated with an increased risk of developing epilepsy after the age of two. The strongest association was observed for PPI, where the risk increased progressively with the number of prescriptions. H2 receptor antagonists were also associated with an increased risk of epilepsy, but their effect was less pronounced compared to PPI, which is compatible with the hypothesis that microbiota alterations may contribute to neurodevelopmental processes, although the available epidemiological studies cannot establish causality (51).

Finally, the possible relationship between prenatal and early childhood exposure to antibiotics and PPI and the risk of childhood cancer was addressed by Gudnadottir et al. The results showed that prenatal exposure to antibiotics and PPI was not significantly associated with the risk of childhood cancer. In contrast, exposure to PPI during the first 2 years of life was associated with an increased risk of cancer occurring after the age of 2 years (aHR approximately 3.68), while antibiotics during the same period did not show a clear association. H2-receptors were excluded from the main analyses due to the very small number of cases. The authors suggest that the critical period for possible effects is early childhood, when the gut microbiome is developing, but emphasize that the results may be influenced by confounding factors (especially the indication for treatment) and that a firm causal relationship cannot be established (52). In conclusion, Table 4 summarizes the main microbiota changes encountered in GERD/GERD and/or treatment with PPI in children.

**TABLE 4:** The main microbiota changes encountered in GERD/GERD and/or treatment with PPI in children.

Author / study	Population/ context	Microbiological Site	Main changes in the microbiota associated with GERD/GERD	Changes associated with PPI treatment	Relevant comments
Duvallet et al. (22)	Children with dysphagia, aspiration and reflux	Oropharynx, stomach, lung	The aerodigestive compartments share common taxa: *Streptococcus*, *Prevotella*, *Haemophilus*, *Veillonella*, *Neisseria*; the pulmonary and gastric microbiome show high inter-individual variability; proximal reflux and full column episodes were moderately correlated with stomach–lung similarity	–	Aspiration was associated with the proximity of the pulmonary microbiome to the oropharyngeal microbiome, without increasing the similarity between the stomach and the lung
Boers et al. (23)	Children with otitis media and a predisposition to reflux	Nasopharynx and middle ear	No significant differences were identified between the microbiota of children with and without reflux; predominance of *Alloiococcus spp.* and *Turicella spp.* in the middle ear	–	There was no translocation of the gastric microbiota or the presence *of Helicobacter pylori*
Harris et al. (24)	Children and adults with GERD, EoE, and controls	Esophagus	GERD Associated with Increased Bacterial Load; Growth *of Firmicutes* and Reduction *of Proteobacteria* in Untreated GERD	PPI → marked expansion of *Proteobacteria* (especially *Aggregatibacter*) and relative reduction of *Streptococcus*	Alpha diversity not significantly changed
Luu et al. (25)	Children with gastroesophageal reflux and esophageal metaplasia	Oral, esophageal, gastric	GER associated with homogenization of oral, esophageal and gastric microbiota; metaplasia associated with the growth of *Prevotella* (especially similar taxon *Prevotella melaninogenica*) and reduction of *Streptococcus*	PPI → Increased Gastric Bacterial Diversity and Increased Contribution of Oral Bacteria to the Stomach	The changes were associated with increased levels of proinflammatory cytokines
Parashette et al. (26)	Healthy children, ER, PPI-REE, EoE	Esophagus	Pathological groups had lower microbial diversity and richness; changes in microbiome composition in RE; reduction in *Vibrionales* in reflux and PPI-REE	–	*Streptococcus*, *Rahnella*, *Leptotrichia*, *Prevotella* involved in microbiota variation
Pantazi et al. (27)	Infants with GER	Intestine	Decrease *Lactobacillus spp.* and *Bacteroides spp.*; growth *of Enterococcus spp.*, *Escherichia coli*, *Klebsiella spp.*	–	Intermediate severity intestinal dysbiosis profile
Ye et al. (28)	Children with GERD	Intestine	Reduced diversity; growth of *Proteobacteria* and *Bacteroidetes*; decrease *of Firmicutes* and *Actinobacteria*; growth *of Bacteroides stercoris*, *B. vulgatus*, *Alistipes putredinis*, *Escherichia-Shigella*, *Klebsiella*, *Haemophilus*; reduction *of Bifidobacterium*, *Faecalibacterium*, *Blautia*, *Lachnospira*	–	Association with metabolic alterations and pro-inflammatory metabolites
Zhang et al. (33)	Children and adolescents treated with PPI	Intestine and oropharynx	–	Intestinal *Streptococcus* increase; decreased *Bifidobacterium*, *Peptostreptococcaceae*, *Turicibacter*; increase of bacteria of oropharyngeal origin in the intestine	No significant changes in global diversity
Rajagopala et al. (34)	Children with normal esophagus exposed to PPI	Esophagus	–	Growth *Streptococcus spp.* and *Prevotella spp.*	No significant changes in alpha/beta diversity
Simakachorn et al. (35)	Children treated 4–8 weeks with PPI	Intestine	–	No major changes in overall diversity or composition; growth trend *Firmicutes* in certain subgroups	Changes dependent on individual characteristics and the environment
Francis et al. (36)	Very young infants with GER treated with PPI	Intestine	–	Increased alpha and beta diversity; growth of taxa from *Firmicutes*; prolonged exposure associated with growth *Flavonifractor*, *Blautia*, *Ruminococcaceae*, *Anaerostipes*, *Lachnospiraceae*; control microbiota dominated by *Bifidobacterium*	Infants treated with PPI showed “accelerated maturation” of the microbiota
Castellani et al. (37)	Infants with GERD assessed before/during/after PPI	Intestine	–	During PPI: decrease *of Lactobacillus* and *Stenotrophomonas*, increase *of Haemophilus*; tendency of growth *of Streptococcus*	The most obvious changes occurred after the discontinuation of PPI, with the maturation of the microbiome
Cares et al. (38)	Children chronically treated with PPI	Small intestine	Possible increased risk of SIBO	–	Statistically insignificant association
Sieczkowska et al. (39)	Children with peptic esophagitis treated with omeprazole	Small intestine	Significant increase in SIBO prevalence after treatment	SIBO-associated PPI and persistent digestive symptoms	Clinically significant association
Hegar et al. (40)	Children treated with omeprazole ± probiotics	Small intestine	About 30% developed SIBO after 1 month of PPI	The probiotics used did not significantly reduce the incidence of SIBO	–
Belei et al. (41)	Children with GERD treated with esomeprazole ± probiotics	Small intestine	SIBO prevalence much lower in the probiotic group	*Lactobacillus reuteri* associated with reduced risk of dysbiosis/SIBO	Protective effect of the probiotic
Dziechciarz et al. (42)	Children with GERD treated with PPI, concomitantly with Lactobacillus rhamnosus GG	It was not directly investigated	No direct microbiological changes associated with GERD/GERD have been reported	No specific microbiotic changes induced by PPI were highlighted; The administration of the probiotic did not reduce the risk of gastrointestinal and respiratory infections associated with antisecretory therapy	The study indirectly assessed the impact of PPI-associated dysbiosis by analyzing infectious risk; The interpretation of the results is limited by the premature interruption of the trial and the reduced statistical power

EoE, eosinophilic esophagitis; GER, gastroesophageal reflux; GERD, gastroesophageal reflux disease; PPI, proton pump inhibitor; PPI-REE, proton pump inhibitor-responsive esophageal eosinophilia; SIBO, small intestinal bacterial overgrowth; spp., species.

## 4 Discussion and limitations

A systematic review of the available literature highlights that the relationship between GERD, PPI use, and alterations in the pediatric microbiome is a field that is still in a relatively early stage of development. The number of available studies is small, and the existing data are marked by significant heterogeneity in terms of the populations investigated, the methods of microbiological analysis, the types of exposures assessed, and the outcomes monitored. However, the results of the included studies convergently suggest the existence of an association between GER, esophageal inflammation, and exposure to antisecretory therapy, on the one hand, and alterations in the aerodigestive and intestinal microbial ecosystems, on the other. Importantly, not all included studies directly characterized the microbiome. Several studies focused on the efficacy, pharmacokinetics, safety, or long-term outcomes of PPI therapy and were included to contextualize the potential clinical implications of microbiota alterations. Consequently, these studies should be regarded as providing indirect rather than direct evidence regarding PPI-associated dysbiosis. From a mechanistic perspective, this association is biologically plausible because the microbiome of the upper and lower aerodigestive tracts is interconnected by anatomical continuity and by physiological or pathological microaspirations. Studies of esophageal microbiome have shown that the esophagus is not a sterile organ, but hosts stable bacterial communities, the structure of which changes significantly in an inflammatory context (53–55).

Current data indicate that GERD is not exclusively a pathology dependent on acid exposure, but may also involve disturbances in local and systemic microbiological homeostasis. Studies focused on the esophageal and upper aerodigestive microbiome have demonstrated that reflux and aspiration were associated with increased similarity between the microbiota of different anatomical compartments, suggesting the existence of a microbial transfer facilitated by repeated exposure to gastric or oropharyngeal contents. In this context, recurrent changes in taxa such as *Streptococcus, Prevotella, Haemophilus* or *Proteobacteria* have been identified, both in the esophagus and at the pulmonary, gastric or intestinal level. At the same time, several studies have suggested that esophageal inflammation associated with GERD or EoE may alter the bacterial load and composition of the local microbiota, even in the absence of major changes in global diversity. This change in bacterial composition is functionally relevant because the increase in the proportion of Gram-negative bacteria may lead to a higher load of lipopolysaccharides (LPS), molecules that activate Toll-like receptors (TLR4) and trigger NF-κB-dependent inflammatory cascades. Activation of these pathways has been associated with the maintenance of chronic esophageal inflammation and progression to Barrett’s esophagus (56–59). In parallel, local inflammation is associated with epithelial barrier dysfunction by altering tight junction proteins (occludin, claudin, ZO-1), which increases mucosal permeability and facilitates the translocation of microbial antigens, amplifying the local immune response (60–62).

At the intestinal level, the included studies frequently describe a dysbiosis profile characterized by a reduction in bacteria considered beneficial, such as *Bifidobacterium, Lactobacillus* or *Faecalibacterium*, concomitant with an increase in taxa associated with inflammation and mucosal permeability, including *Enterococcus, Escherichia-Shigella, Klebsiella* or various species of *Proteobacteria*. Metabolomic analyses further suggest that these microbial alterations may influence metabolic pathways involved in inflammation, energy metabolism and immunological regulation, supporting the hypothesis of a complex interaction between the microbiota, the immune system and the pathophysiology of GERD. Functionally, one of the major consequences of this dysbiosis is the reduction in the production of SCFAs, especially butyrate, which is an essential metabolite for intestinal homeostasis. Butyrate supports the integrity of the epithelial barrier, induces the differentiation of regulatory T lymphocytes, and limits inflammation by inhibiting HDACs and regulating inflammatory gene expression (63–65). Furthermore, the decrease in SCFAs-producing bacteria such as *Faecalibacterium prausnitzii* is associated with the loss of mucosal anti-inflammatory effects and increased susceptibility to chronic low-grade inflammation (66, 67).

A central aspect highlighted by most of the studies reviewed is the impact of PPI therapy on the pediatric microbiome. Although PPI remain the main pharmacological treatment used for symptom control and acid suppression, numerous data suggest that reducing gastric acidity can significantly alter the microbial ecology of the upper and lower digestive tract. Mechanistically, gastric acidity represents a major barrier against bacterial colonization. Its reduction by PPI has been proposed to promote the phenomenon of “oralization of gut microbiome,” in which oral bacteria survive gastric transit and colonize the distal intestine (68–70). Several longitudinal studies have shown that PPI use has been associated with increased survival and translocation of oropharyngeal bacteria to the stomach and intestine, with a consequent increase in taxa such as *Streptococcus* and a decrease in protective bacteria such as *Bifidobacterium*. However, the results regarding the magnitude of these changes are inconsistent, with some studies reporting only spot taxonomic changes, without significant alterations in overall diversity. These discrepancies may reflect important methodological differences between studies, including duration of treatment, age of patients, type of diet, antibiotic exposure, and stage of maturation of the infant microbiome. Variations are also explainable in terms of the difference in the maturation of the pediatric microbiome, the child’s microbiota being much more plastic and influenced by factors such as type of birth, nutrition, and exposure to antibiotics (19, 20).

In parallel, several studies have raised the question of the potential clinical consequences of PPI-associated dysbiosis. The association between chronic PPI use and SIBO has been repeatedly described, although the level of evidence remains variable. From a pathophysiological perspective, the reduction in gastric acidity is expected to reduce the main mechanism for sterilizing ingested contents, facilitating colonization of the small intestine with bacteria of oral and colonic origin. In addition, PPI-induced hypochlorhydria may alter intestinal motility and promote luminal stasis, creating an environment conducive to excessive bacterial proliferation. This combination of mechanistic factors supports the biological plausibility of the association between PPI and SIBO, even though the magnitude of the reported risk varies between studies (71–74). In addition, data from large population-based cohorts suggest associations between early exposure to PPI and an increased risk of gastrointestinal and respiratory infections, atopy, neuropsychiatric disorders, and other long-term pathologies. These observations are consistent with the central role of the gut microbiome in the maturation of the immune system, particularly during early life, when microbial colonization contributes to the development of immunological tolerance and the calibration of the inflammatory response. Disruption of this process by reducing microbial diversity and altering the production of immunomodulatory metabolites (particularly SCFAs) may be associated with to a dysregulated immune phenotype characterized by increased susceptibility to infections and exaggerated allergic responses. Furthermore, the gut–brain axis provides a biological framework for possible associations with neuropsychiatric disorders, through microbial metabolites, modulation of systemic inflammation, and interaction with the enteric and central nervous systems (75–77). However, it is essential to emphasize that most of these associations come from observational studies, which introduces a significant risk of residual confounding. The indication for PPI may itself be a marker of the severity of the underlying disease or of comorbidities that independently contribute to infectious or allergic risk. In addition, factors such as concomitant antibiotic exposure, frequent hospitalizations, diet, and socioeconomic status may influence both the decision to prescribe PPI and the composition of the microbiome, complicating causal inference.

Regarding therapeutic interventions, the control of the use of probiotics as a strategy to prevent or correct PPI-induced dysbiosis is still inconclusive. Some clinical trials have reported a reduction in the prevalence of SIBO and improvement in digestive symptoms with concomitant administration of probiotics, suggesting a potential role in restoring microbial balance and ecological competition against opportunistic bacteria. However, other studies have not identified significant benefits, and existing meta-analyses highlight a significant heterogeneity of results. This variability can be explained by major differences in the composition of probiotics, as the effects are strongly strain-specific and cannot be generalized at the “class” level. Also, the duration of the intervention, the timing of administration relative to PPI initiation, and host characteristics (age, baseline microbiome, diet) may significantly influence the clinical response. Furthermore, it is possible that the effects of probiotics are modulatory rather than corrective, having limited impact under conditions of persistent ecological disturbance induced by chronic acid suppression (78–83).

The overall interpretation of the results must be made in the context of important methodological limitations of the available literature. Most studies are observational, single-center, and include small samples, without the possibility of fully controlling for confounding factors such as diet, mode of delivery, prematurity, antibiotic use, or the exact indication for antisecretory therapy. In addition, the heterogeneity of microbiological analysis techniques and the lack of methodological standardization limit the comparability of results and the generalizability of the findings. To provide a structured assessment of the certainty of the evidence, we performed a GRADE-based evaluation of the main outcome domains (Table 5). Certainty was assessed across the domains of risk of bias, inconsistency, indirectness, and imprecision. Overall, the certainty of evidence was rated as very low for GERD-associated microbiota alterations and PPI-associated microbiota alterations, reflecting the predominance of observational studies, considerable heterogeneity, and substantial risk of confounding. Evidence regarding PPI exposure and SIBO/dysbiosis-related small bowel alterations was judged as low certainty, while outcomes related to PPI efficacy, pharmacokinetics, allergic complications, and infectious outcomes reached moderate certainty, primarily due to the inclusion of randomized clinical trials or large population-based cohort studies. Finally, evidence linking PPI exposure to neuropsychiatric, epilepsy, and cancer-related outcomes was rated as low certainty, mainly because of the observational nature of the available studies and concerns regarding indirectness and residual confounding. Overall, current evidence supports the existence of a complex relationship between GERD, PPI therapy, and alterations of the pediatric microbiome; however, the precise pathophysiological mechanisms and their long-term clinical implications remain insufficiently established. Future prospective multicenter studies with larger pediatric cohorts, standardized microbiome assessment protocols, longitudinal follow-up, and integration of multi-omics approaches are needed to clarify the causal relationship between dysbiosis and reflux disease and to better define the long-term safety profile of antisecretory therapy during childhood.

**TABLE 5:** GRADE assessment of the certainty of evidence across the main clinical and microbiome-related outcomes.

Outcome	Included studies	Design	Risk of bias	Inconsistency	Indirectness	Imprecision	Overall certainty
GERD-associated microbiota alterations	Duvallet et al. (22); Boers et al. (23); Harris et al. (24); Luu et al. (25); Parashette et al. (26); Pantazi et al. (27) Ye et al. (28)	Observational studies (cohort, cross-sectional, case-control)	Serious	Serious	Serious	Serious	Very low
PPI-associated microbiota alterations	Zhang et al. (33); Rajagopala et al. (34); Simakachorn et al. (35); Francis et al. (36); Castellani et al. (37)	Prospective observational cohorts / before–after studies	Serious	Serious	Serious	Serious	Very low
PPI exposure and SIBO/dysbiosis-related small bowel alterations	Cares et al. (38); Sieczkowska et al. (39); Hegar et al. (40); Belei et al. (41); Dziechciarz et al. (42)	Randomized trials + observational studies	Serious	Moderate	Moderate	Serious	Low
PPI efficacy, pharmacokinetics and tolerability in pediatric GERD	Zhao et al. (29); Gilger et al. (30); Winter et al. (31); Omari et al. (32)	Randomized clinical trials / pharmacokinetic studies	Some concerns	Moderate	Low	Moderate	Moderate
PPI exposure and allergic outcomes	Mitre et al. (43); Chaaban et al. (44)	Large retrospective population-based cohorts	Moderate	Low	Moderate	Moderate	Moderate
PPI exposure and infectious outcomes	Lassalle et al. (45); van der Sande et al. (47); Freedberg et al. (48); Bîrluţiu et al. (46)	Population-based cohorts + case report	Moderate	Low	Moderate	Moderate	Moderate
PPI exposure and neuropsychiatric outcomes	Stark et al. (49); Wang et al. (50)	Retrospective population-based cohorts	Serious	Moderate	Serious	Moderate	Low
PPI exposure and epilepsy/cancer outcomes	Gudnadottir et al. (51); Gudnadottir et al. (52)	Nationwide population-based cohorts	Moderate	Low	Serious	Moderate	Low

GERD, gastroesophageal reflux disease; PPI, proton pump inhibitor; SIBO, small intestinal bacterial overgrowth.

A limitation of this review is that it intentionally integrates evidence derived from both disease-associated and treatment-associated microbiota alterations. Although these represent conceptually distinct exposures, they are biologically interconnected in pediatric GERD because acid suppression therapy is part of routine disease management. For this reason, the evidence was synthesized within a predefined conceptual framework rather than as separate systematic reviews. At the same time, although several studies evaluated related outcomes such as SIBO, infections, or allergic diseases, the small number of studies available for each outcome, together with differences in study design, diagnostic criteria, exposure definitions, follow-up periods, and reported effect measures, precluded meaningful quantitative pooling. Consequently, a narrative synthesis was considered the most appropriate approach. Nevertheless, residual confounding by indication should be considered when interpreting studies evaluating PPI-associated outcomes. Another limitation is that the literature search was restricted to PubMed, Scopus, and Web of Science. Although these databases cover a large proportion of the biomedical literature, relevant studies indexed exclusively in databases such as Embase, Cochrane Library, or CINAHL may have been missed.

## 5 Conclusion

The present systematic review indicates that the relationship between GERD, PPI exposure, and alterations of the pediatric microbiome is an emerging field characterized by a limited number of studies, substantial methodological heterogeneity, and an overall low certainty of evidence. Differences in study populations, microbiological sites, analytical methods, and exposure definitions limit direct comparison across studies. The available evidence suggests that GER/GERD, aspiration, and mucosal inflammation are associated with microbiome alterations throughout the aerodigestive tract, including changes in bacterial diversity and composition, as well as increased similarity between microbial communities from different anatomical compartments, supporting the possibility of reflux- or aspiration-mediated microbial transfer. However, this phenomenon appears heterogeneous, and it remains unclear whether these microbiota alterations represent a cause, a consequence, or an adaptive response to inflammation. PPI therapy has also been associated with microbiome remodeling, including increased abundance of oral bacteria in the lower gastrointestinal tract, changes in microbial diversity, alterations in the *Firmicutes–Bacteroidetes* ratio, and reductions in beneficial taxa such as *Bifidobacterium* and, in some studies, *Lactobacillus*. Nevertheless, these findings remain inconsistent and are likely influenced by age, treatment duration, underlying disease, diet, antibiotic exposure, and other environmental factors. Likewise, evidence supporting an association between PPI therapy and SIBO remains limited and heterogeneous. Beyond the gastrointestinal tract, observational studies have reported associations between early PPI exposure and an increased risk of infections, allergic diseases, neuropsychiatric disorders, and other long-term adverse outcomes. However, these findings should be interpreted cautiously because they derive predominantly from observational studies and remain susceptible to residual confounding and indication bias. Overall, the microbiome represents a biologically plausible mediator linking GERD, PPI therapy, and local or systemic effects during childhood. However, according to the GRADE assessment performed in this review, the certainty of evidence for most microbiome-related outcomes is low or very low. Consequently, current evidence supports biological associations rather than causal relationships. Future well-designed prospective, longitudinal, multicenter studies using standardized microbiome methodologies and rigorous control of confounding factors are needed to clarify the causal role and clinical significance of microbiome alterations in pediatric GERD and during PPI therapy.

## Data Availability

The original contributions presented in this study are included in this article/supplementary material, further inquiries can be directed to the corresponding author.
